# Nationwide Spatial Patterns and Maternal and Birth-Related Factors Associated with Orofacial Clefts in Brazil

**DOI:** 10.3390/ijerph22070995

**Published:** 2025-06-24

**Authors:** Luis Gustavo Souza Santos, Vandilson Rodrigues, Jessilene Ribeiro Rocha, Mila Roselaine Lima de Assunção, Marcio Vinícius Campos Borges, Maria Carmen Fontoura Nogueira da Cruz

**Affiliations:** 1 Dentistry Graduate Program, Federal University of Maranhão, São Luís 65085-580, Brazil; souza.luis@discente.ufma.br (L.G.S.S.); vandilson.rodrigues@ufma.br (V.R.); jessilene.rr@discente.ufma.br (J.R.R.); mila.assuncao@discente.ufma.br (M.R.L.d.A.); marcio.borges@discente.ufma.br (M.V.C.B.); 2 Department of Morphology, Federal University of Maranhão, São Luis 65085-580, Brazil

**Keywords:** orofacial clefts, epidemiology, spatial analysis, Brazil

## Abstract

This study aimed to identify spatial clustering and maternal and birth-related factors associated with the incidence of orofacial clefts in Brazil from 2001 to 2022. A nationwide ecological study was conducted in Brazil using data from 2001 to 2022 obtained from the Brazilian Live Birth Information System (SINASC). The municipality was used as the spatial unit of analysis. Variables included maternal age and education, newborn sex, gestational age, birth weight, and skin color/ethnicity. Univariate and bivariate global and local Moran’s I indices were used to assess spatial autocorrelation. A total of 234 municipalities (4.2%) formed high–high spatial clusters, primarily in the South and Southeast, while 431 municipalities (7.7%) formed low–low clusters, mostly in the Northeast (Moran’s I = 0.121, 95% CI: 0.107 to 0.135). High–high clusters had a lower median proportion of adolescent mothers (≤19 years: 17.4%) and a higher proportion of mothers aged ≥ 35 years (12.9%) compared to low–low clusters (23.5% and 8.7%, respectively; *p* < 0.001). High–high clusters also had fewer mothers with less than seven years of education (31.0% vs. 45.9%, *p* < 0.001) and higher rates of preterm births and low birth weight (*p* < 0.001). The proportion of White newborns was higher in high–high clusters than in low–low clusters (82.8% vs. 13.6%, *p* < 0.001). These findings suggest that orofacial cleft incidence in Brazil is spatially associated with maternal sociodemographic characteristics, perinatal outcomes, and newborn race/ethnicity.

## 1. Introduction

Orofacial clefts are congenital malformations that result from disruptions in the embryological processes responsible for the fusion of facial prominences during fetal development [1]. These anomalies may affect the lip, the palate, or both and can vary in laterality and severity [2]. Although the etiology of orofacial clefts is not fully understood, they are widely recognized as having a multifactorial origin, involving both genetic and environmental factors. Current evidence suggests that genetic predisposition is the primary determinant, with environmental factors, including nutritional deficiencies, smoking, alcohol consumption, and drug use during pregnancy, acting as modulators that can increase the likelihood or severity of cleft formation [1,2,3].

These malformations are frequently associated with both functional and aesthetic complications [4]. Affected individuals often face challenges related to speech, feeding, and craniofacial development [5]. Additionally, the social stigma associated with orofacial clefts can lead to emotional distress and difficulties in social integration. As a result, comprehensive management of these conditions requires a multidisciplinary approach involving professionals from various fields, such as dentistry, speech therapy, plastic surgery, and psychology [6,7,8].

Understanding the factors associated with the occurrence of orofacial clefts is essential for developing effective prevention strategies and improving healthcare delivery [9]. Previous studies in Brazil have primarily focused on specific regions or conducted analyses at the state level [10,11,12,13,14,15,16], revealing differences in orofacial cleft rates and temporal trends across states. However, no prior study has investigated spatial clustering at the municipal level, which offers a more detailed view of geographic patterns. This is particularly important in a demographically diverse country like Brazil, where local-level analysis can more accurately inform targeted public health interventions.

Brazil is the fifth largest country in the world by land area (~8.5 million km^2^) and had an estimated population of over 212 million in 2024 [17]. Administratively, it is divided into 26 states and a Federal District, comprising more than 5500 municipalities. Thus, there remains a gap in nationwide, municipality-level investigations. Moreover, spatial analyses can offer valuable insights to guide public health officials in designing more effective and culturally sensitive policies. To address existing knowledge gaps, this study aims to identify spatial clusters of municipalities with high and low incidence rates of live births with orofacial clefts and to examine their association with maternal and birth-related factors using data from Brazil’s Live Birth Information System (SINASC).

## 2. Materials and Methods

### 2.1. Study Design

This study employed a nationwide ecological observational design, with each Brazilian municipality serving as the spatial unit of analysis. Data were extracted from the Live Birth Information System (SINASC) [18], maintained by the Health Surveillance Secretariat of the Brazilian Ministry of Health. The analysis covered the period from 2001 to 2022.

### 2.2. Data Source

This study utilized secondary data obtained from SINASC [18], a national surveillance system that provides comprehensive information on all live births across Brazil. This open-access database system is based on data recorded in the Live Birth Certificate (Declaração de Nascido Vivo, in Portuguese), a mandatory document for every live birth in the country. SINASC ensures standardized data collection across all levels of healthcare and plays a critical role in informing maternal and child health policies, as well as in supporting surveillance and planning efforts. Monitoring live birth statistics through SINASC is essential for effective health management, as it provides valuable insights into population health conditions and helps guide the development and implementation of maternal and child health strategies.

### 2.3. Data Collection

The study included data from all Brazilian municipalities (*n* = 5570). The dependent variable was the rate of live births with orofacial clefts, calculated for each Brazilian municipality. This rate was expressed as the number of live births with orofacial clefts per 1000 live births during the period from 2001 to 2022.

The independent variables consisted of sociodemographic characteristics related to both the mother and the newborn. Maternal variables included the following: (a) age group, measured as the percentage of mothers aged ≤ 19 years and >35 years, and (b) low educational level, defined as the percentage of mothers with fewer than seven years of formal education. Newborn-related variables included the following: (a) race/ethnicity, categorized as White, Black, Asian, Brown (Pardo, in Portuguese), and Indigenous; (b) preterm birth, expressed as the percentage of births occurring before 37 weeks of gestation; and (c) low birth weight, defined as the percentage of newborns weighing less than 2500 g at birth. All variables were calculated as proportions of total live births in each municipality over the study period. The use of aggregated data allowed for spatial pattern analysis and regional comparison of associated factors.

### 2.4. Statistical and Spatial Analysis

Descriptive statistics were used to summarize the incidence of orofacial clefts and maternal and birth-related variables across Brazilian municipalities from 2001 to 2022. The incidence rate of orofacial clefts was calculated as the number of live births with orofacial clefts per 1000 live births, stratified by municipality and region. Median and interquartile ranges (IQR) were used to describe continuous variables with non-normal distribution. Comparative analyses between municipalities classified as high–high and low–low spatial clusters were conducted using the Mann–Whitney U test for non-parametric data. A significance level of *p* < 0.05 was adopted for all statistical tests.

Spatial analysis was conducted using Brazilian municipalities as the spatial unit of aggregation. A univariate Global Moran’s I statistic was calculated to assess the overall spatial autocorrelation of orofacial cleft incidence across municipalities. To identify localized spatial clusters, Local Indicators of Spatial Association (LISA) were computed using the univariate and bivariate Local Moran’s I statistics. The univariate LISA classified municipalities into five categories based on spatial relationships: high–high (hot spots), low–low (cold spots), high–low, low–high (spatial outliers), and not significant. Bivariate LISA analyses were used to explore spatial associations between orofacial cleft incidence and maternal and birth-related variables, including maternal age, education level, number of prenatal visits, gestational age, birth weight, and newborn race/ethnicity.

All spatial analyses were performed using GeoDa software version 1.16.0.12. The spatial weights matrix was constructed based on contiguity (first-order queen criterion), and significance was assessed using 999 random permutations. Thematic maps, including incidence rates and LISA cluster maps, were created using QGIS software version 2.18 to visually represent spatial distribution and cluster patterns of orofacial clefts across Brazilian municipalities.

## 3. Results

**Figure 1:**
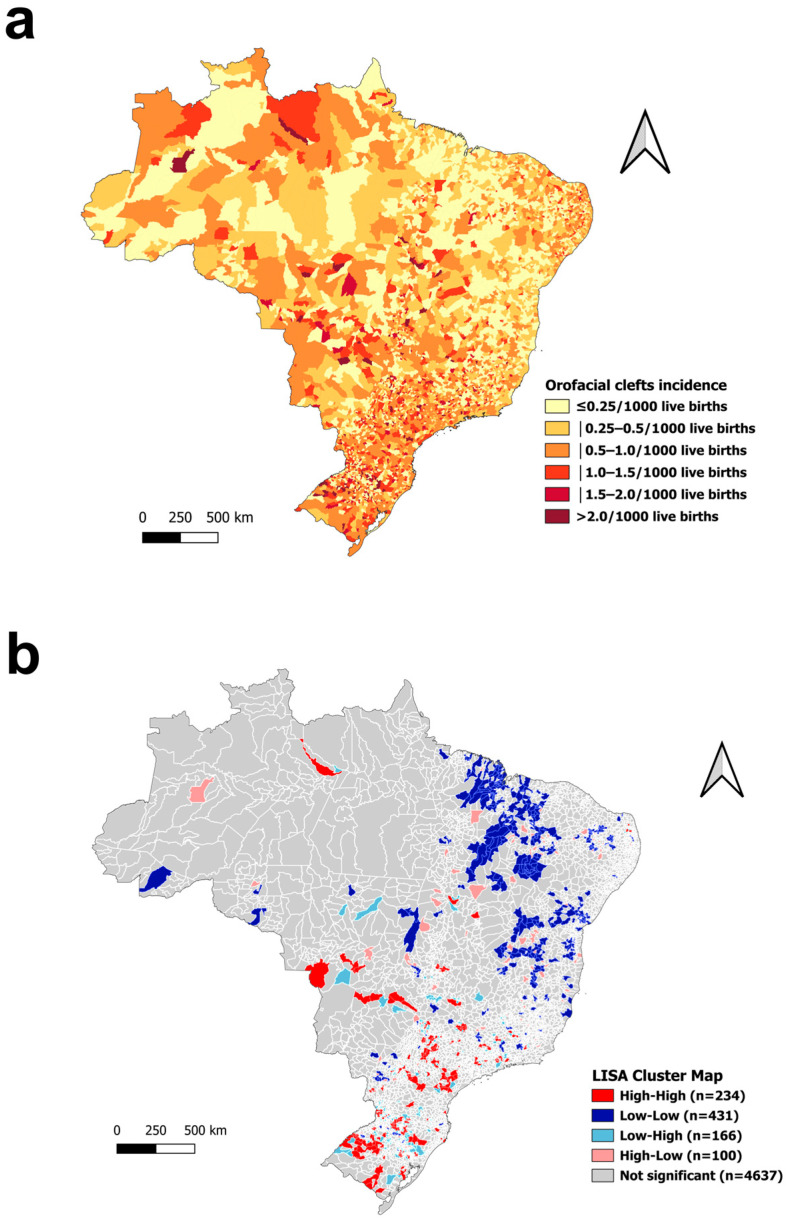
Spatial distribution of orofacial cleft incidence in Brazil: incidence rates per 1000 live births by municipality (**a**) and Local Indicators of Spatial Association (LISA) cluster map (**b**).

This study included data on 64,053,752 live births in Brazil between 2001 and 2022. During the study period, the overall rate of live births with orofacial clefts in Brazil was 0.51 per 1000 live births. The majority of municipalities in all regions reported relatively low incidence rates of orofacial clefts. Nationally, 37.3% of municipalities had rates ≤ 0.25/1000 live births. The South and Southeast regions had higher proportions of municipalities with elevated incidence rates. For example, 6.2% of municipalities in the South and 2.6% in the Southeast reported rates above 2.0/1000 live births. In contrast, the Northeast region had the highest concentration of municipalities with the lowest incidence rates, with 45.1% recording ≤0.25 per 1000 live births and only 0.2% exceeding 2.0 per 1000 (Table 1, Figure 1a).

A total of 234 municipalities (4.2%) were classified as high–high clusters, indicating areas with high incidence rates surrounded by municipalities with similarly high rates. These were predominantly located in the South (10.5%) and Southeast (5.3%). The Northeast had the largest number of low–low clusters (17.1%), representing municipalities with low incidence rates surrounded by others with similarly low rates. Low–high and high–low outliers were most commonly found in the South, suggesting spatial heterogeneity within this region (Table 1, Figure 1b). The global univariate Moran’s I index for orofacial cleft incidence was 0.121 (95% CI = 0.107 to 0.135, *p* < 0.001), indicating a statistically significant, though modest, positive spatial autocorrelation.

Table 2 presents a comparative analysis between municipalities classified as high–high and low–low spatial clusters of orofacial cleft incidence, along with the corresponding bivariate Moran’s I values indicating spatial correlation. Municipalities in the high–high cluster exhibited a significantly lower median percentage of adolescent mothers (≤19 years: 17.4%) and a higher percentage of mothers aged ≥ 35 years (12.9%) compared to the low–low cluster (23.5% and 8.7%, respectively, *p* < 0.001). High–high clusters also had fewer mothers with less than seven years of education (31.0% vs. 45.9%, *p* < 0.001). Spatial analysis revealed a negative spatial autocorrelation between orofacial cleft rates and both adolescent motherhood (Moran’s I = −0.162, 95% CI = −0.174 to −0.150) and low maternal education (Moran’s I = −0.155, 95% CI = −0.167 to −0.143). In contrast, a positive spatial correlation was observed with the proportion of mothers aged ≥ 35 years (Moran’s I = 0.164, 95% CI = 0.152 to 0.176), suggesting that areas with higher orofacial cleft incidence tend to cluster around municipalities with more older mothers.

Regarding birth outcomes (Table 3), high–high municipalities reported higher rates of preterm births (8.9%) and low-birth-weight newborns (8.5%) compared to low–low clusters (7.5% and 6.9%, respectively, *p* < 0.001). Positive spatial correlations were also found between orofacial cleft incidence and both preterm birth (Moran’s I = 0.084) and low birth weight (Moran’s I = 0.113), indicating spatial clustering of these adverse outcomes. In terms of newborn race/ethnicity, the proportion of White newborns was markedly higher in high–high clusters (82.8%) than in low–low clusters (13.6%), while the opposite trend was observed for Brown (13.2% vs. 72.9%) and Black (1.9% vs. 3.4%) newborns. Correspondingly, orofacial cleft incidence showed a strong positive spatial correlation with the percentage of White newborns (Moran’s I = 0.257, 95% CI = 0.243 to 0.270) and negative correlations with the percentages of Black (Moran’s I = −0.071, 95% CI = −0.082 to −0.060) and Brown (Moran’s I = −0.244, 95% CI = −0.255 to −0.233) newborns. No significant spatial correlation was found with Indigenous ethnicity (Moran’s I = 0.001, 95% CI = −0.010 to 0.012).

## 4. Discussion

The main findings of this nationwide study suggest that a range of sociodemographic and perinatal factors are associated with the incidence of orofacial clefts in Brazil between 2001 and 2022. Spatial analysis revealed substantial geographic heterogeneity in case distribution, with higher incidence rates concentrated in the Southern region and lower rates in the Northeast. These results are consistent with previous Brazilian studies [10,13,16,19,20] and may reflect regional differences in genetic ancestry and healthcare system performance. The South and Southeast regions are predominantly composed of individuals of White ancestry, a group reported to have a higher prevalence of orofacial clefts. In contrast, the North and Northeast, characterized by larger populations of Black and Brown (Pardo) individuals, have historically shown lower incidence rates. Additionally, underreporting, particularly in socioeconomically disadvantaged and geographically remote areas, likely contributes to these discrepancies [21].

Although the North and Northeast exhibited lower average incidence rates, previous studies have reported increasing trends in these regions [13,16,19]. These trends may be linked to changing exposures to risk factors, including environmental teratogens and maternal lifestyle behaviors. Moreover, improvements in Brazil’s Unified Health System (Sistema Único de Saúde, in Portuguese), such as expanded healthcare coverage, enhanced surveillance systems, and improved training of healthcare personnel, may have contributed to more accurate reporting and a reduction in underreporting of congenital anomalies over time [22].

Regarding maternal characteristics, the present study found a negative spatial association between adolescent motherhood (≤19 years) and orofacial cleft incidence, suggesting that younger maternal age was not a significant risk factor. This finding aligns with a study conducted in Northeastern Brazil, which showed that only 15% of orofacial cleft cases occurred in infants born to adolescent mothers [23]. However, studies from other regions report conflicting results. For example, a case-control study in China found that adolescent mothers had a higher likelihood of giving birth to infants with orofacial clefts [24]. Similarly, data from the Birth Defects Surveillance System in Hunan Province, China, also identified young maternal age as a potential risk factor [25]. These discrepancies highlight the complex and multifactorial etiology of orofacial clefts and suggest that the influence of maternal age may be modulated by contextual variables such as genetics, nutrition, and access to healthcare services.

In contrast, advanced maternal age (≥35 years) was positively associated with higher orofacial cleft incidence in the present study. This association is consistent with previous findings [13,26]. Older maternal age is known to increase the risk of chromosomal abnormalities and fetal developmental complications. Potential biological mechanisms underlying this association include oocyte aging, chronic maternal conditions, and prolonged exposure to environmental teratogens. In addition, changes in gamete quality, altered placental permeability, and medication use during pregnancy may contribute to the heightened risk of congenital anomalies in older mothers [16,27].

Maternal education level also emerged as an important factor. A lower percentage of mothers with fewer than seven years of schooling was observed in high–high clusters of orofacial cleft incidence compared to low–low clusters, suggesting a negative association. However, previous studies have reported that lower maternal education is associated with higher rates of orofacial cleft occurrence [24,28,29]. A Brazilian case-control study also reported a higher prevalence of orofacial clefts among families with lower educational attainment. However, this study was conducted in only two hospitals in Cuiabá (Mato Grosso state) and included a small sample size (40 children affected by orofacial clefts and 40 controls), which limits the generalizability of its findings [30]. This apparent contradiction may be partially explained by limitations of the SINASC system, which includes only data on live births. Since low maternal education is associated with increased neonatal mortality, stillbirths, and early neonatal deaths among infants with orofacial clefts may be underreported. As a result, this underrepresentation could contribute to lower observed incidence rates in more socioeconomically vulnerable populations [31].

Perinatal outcomes such as preterm birth and low birth weight were strongly associated with higher orofacial cleft incidence, consistent with previous studies [19,32,33]. One biological explanation for this association is the common scheduling of cesarean deliveries for infants diagnosed with orofacial clefts, which may result in births occurring before the optimal gestational age for fetal weight gain. Additionally, orofacial clefts themselves may contribute to intrauterine growth restriction or be part of syndromic conditions that predispose affected infants to adverse perinatal outcomes. Studies conducted in the United States have indicated that advanced maternal age [32], maternal diabetes, and hypertension [34], as well as low birth weight and prematurity [33], are associated with an increased risk of orofacial clefts, supporting the multifactorial nature of these congenital malformations.

Significant disparities were also observed regarding race/ethnicity. The incidence of orofacial clefts was higher in municipalities with a greater proportion of White newborns and lower in areas with larger populations of Brown (Pardo) and Black newborns. These findings align with global trends indicating a higher prevalence of orofacial clefts among populations of European descent [35,36]. A previous meta-analysis focused on populations of European ancestry identified statistically significant associations between non-syndromic orofacial clefts and 19 genetic variants located in or near 13 genes/loci, including IRF6, GRHL3, 8q24, VAX1, TGFA, FOXE1, ABCA4, NOG, GREM1, AXIN2, DVL2, WNT3A, and WNT5A [37]. These variants were consistently associated with different non-syndromic orofacial cleft phenotypes under various genetic models, underscoring their potential role as biomarkers in European populations. This evidence provides strong biological plausibility for the higher prevalence of orofacial clefts observed among individuals of European ancestry, including those living in Brazil’s South and Southeast regions, where this ancestry is more prominent [38]. In the present study, no significant association was found with Indigenous ethnicity. The variability in these results may reflect Brazil’s high degree of racial admixture, genetic diversity, and inconsistent classification of race/ethnicity in official records.

This study has some limitations. Underreporting remains the most critical constraint, particularly in remote and low-resource municipalities, where data completeness and diagnostic accuracy are compromised. Throughout the study period, several municipalities had missing or inconsistent records, raising concerns about data reliability. The accuracy of the data depends on the training and attentiveness of healthcare personnel involved in birth reporting. Furthermore, the ecological design limits causal inference and may obscure individual-level relationships. Moreover, the study did not incorporate potentially relevant factors such as genetic predisposition, environmental exposures, healthcare infrastructure, or geographic features. Future studies integrating these variables, along with genomic and syndromic classification data, are warranted to gain a more comprehensive understanding of cleft etiology in Brazil.

Another limitation of the study is related to the classification of race/ethnicity in the Brazilian SINASC database. These data are based on self-identification and may lead to misclassification or inconsistent reporting across regions and over time. Due to Brazil’s significant racial admixture and the subjective nature of racial categories [39,40], particularly among those identifying as Brown (Pardo), spatial correlations involving race/ethnicity should be interpreted with caution. Moreover, factors such as genetic predisposition and exposure to environmental teratogens may also influence the occurrence of orofacial clefts. Therefore, future research incorporating genetic, environmental, and ancestry-informed approaches would enhance the accuracy and depth of epidemiological analyses.

Despite these limitations, the study presents several strengths. It is the first to conduct a nationwide spatial analysis of orofacial clefts using live birth records from all Brazilian municipalities over two decades. The use of spatial statistical techniques, including Global Moran’s I and Local Indicators of Spatial Association (LISA), enabled the identification of high-risk clusters and their correlates. This approach provides a valuable framework for health policy and planning by allowing the geographic targeting of preventive efforts and resource allocation to underserved regions.

Identifying spatial clusters of high and low incidence can support a more targeted approach to surveillance, prevention, and early intervention. These results underscore the importance of improving data completeness and quality within national health information systems and integrating sociodemographic considerations into policies aimed at reducing congenital anomalies. The present study can provide a robust foundation for future research and policy initiatives to address regional disparities and improve outcomes for individuals affected by orofacial clefts in Brazil.

## 5. Conclusions

The findings of this study suggest that sociodemographic and perinatal factors are spatially associated with the incidence of orofacial clefts in Brazil. Specifically, maternal age ≥ 35 years, low birth weight (<2500 g), prematurity, and White race/ethnicity demonstrated positive geographic correlations with orofacial cleft occurrence. These spatial associations highlight the need for context-sensitive public health interventions tailored to the specific profiles of different regions.

## Figures and Tables

**Table 1:** Distribution of orofacial cleft incidence rates and spatial autocorrelation cluster categories by Brazilian geographic region.

Variables	Central-West		Northeast		North		Southeast		South		Overall	
	*n*	%	*n*	%	*n*	%	*n*	%	*n*	%	*n*	%
Incidence rate (per 1000 live births)												
≤0.25	163	34.9	809	45.1	173	38.4	558	33.5	374	31.4	2077	37.3
|0.25–0.5	94	20.1	488	27.2	124	27.6	325	19.5	129	10.8	1160	20.8
|0.5–1.0	127	27.2	394	22.0	116	25.8	510	30.6	370	31.1	1517	27.2
|1.0–1.5	50	10.7	81	4.5	26	5.8	171	10.3	169	14.2	497	8.9
|1.5–2.0	19	4.1	18	1.0	5	1.1	61	3.7	75	6.3	178	3.2
>2.0	14	3.0	4	0.2	6	1.3	43	2.6	74	6.2	141	2.5
LISA categories												
High–High	13	2.8	3	0.2	4	0.9	89	5.3	125	10.5	234	4.2
Low–Low	10	2.1	306	17.1	20	4.4	71	4.3	24	2.0	431	7.7
Low–High	11	2.4	3	0.2	3	0.7	51	3.1	98	8.2	166	3.0
High–Low	7	1.5	49	2.7	7	1.6	25	1.5	12	1.0	100	1.8
Not significant	426	91.2	1433	79.9	416	92.4	1432	85.9	932	78.3	4637	83.2

LISA = Local Indicators of Spatial Association.

**Table 2:** Comparative analysis and spatial correlation of maternal factors between high–high and low–low clusters of orofacial cleft incidence in Brazil.

Factors (Percentage by Municipality)	LISA Clusters of Orofacial Clefts Incidence				*p*-Value	Moran’s I (95% CI)
	High–High		Low–Low			
	Median	(IQR)	Median	(IQR)		
Maternal factors						
Aged ≤ 19 years	17.4	(6.2)	23.5	(8.6)	<0.001	−0.162 (−0.174 to −0.150)
Aged ≥ 35 years	12.9	(4.3)	8.7	(4.2)	<0.001	0.164 (0.152 to 0.176)
Up to 7 years of education	31.0	(12.9)	45.9	(14.3)	<0.001	−0.155 (−0.167 to −0.143)

IQR = interquartile range. Moran’s I = Global bivariate Moran’s index. 95% CI = 95% confidence interval.

**Table 3:** Comparative analysis and spatial correlation of birth-related factors between high–high and low–low clusters of orofacial cleft incidence in Brazil.

Factors (Percentage by Municipality)	LISA Clusters of Orofacial Clefts Incidence				*p*-Value	Moran’s I (95% CI)
	High–High		Low–Low			
	Median	(IQR)	Median	(IQR)		
Birth-related factors						
Preterm birth	8.9	(1.8)	7.5	(2.0)	<0.001	0.084 (0.073 to 0.095)
Low birth weight	8.5	(1.5)	6.9	(14.3)	<0.001	0.113 (0.102 to 0.124)
Newborn race/ethnicity						
White	82.8	(17.2)	13.6	(20.1)	<0.001	0.257 (0.243 to 0.270)
Black	1.9	(2.1)	3.4	(3.4)	<0.001	−0.071 (−0.082 to −0.060)
Brown (Pardo)	13.2	(16.0)	72.9	(26.4)	<0.001	−0.244 (−0.255 to −0.233)
Indigenous	0.03	(0.11)	0.13	(0.17)	0.829	0.001 (−0.010 to 0.012)

IQR = interquartile range. Moran’s I = Global bivariate Moran’s index. 95% CI = 95% confidence interval.

## Data Availability

The databases used in this study are open source and made publicly available by the Brazilian government at http://tabnet.datasus.gov.br (accessed on 10 January 2025).

## Abbreviations

The following abbreviations are used in this manuscript:

IQR	Interquartile range
LISA	Local Indicators of Spatial Association
SINASC	Brazilian Live Birth Information System
SUS	Brazil’s Unified Health System

## References

[1] Hammond N.L., Dixon M.J. (2022). Revisiting the embryogenesis of lip and palate development. Oral Dis..

[2] Nasreddine G., El Hajj J., Ghassibe-Sabbagh M. (2021). Orofacial clefts embryology, classification, epidemiology, and genetics. Mutat. Res. Rev. Mutat. Res..

[3] Fonseca-Souza G., de Oliveira L.B., Wambier L.M., Scariot R., Feltrin-Souza J. (2022). Tooth abnormalities associated with non-syndromic cleft lip and palate: Systematic review and meta-analysis. Clin. Oral Investig..

[4] Salari N., Darvishi N., Heydari M., Bokaee S., Darvishi F., Mohammadi M. (2022). Global prevalence of cleft palate, cleft lip and cleft palate and lip: A comprehensive systematic review and meta-analysis. J. Stomatol. Oral Maxillofac. Surg..

[5] Pereira V.J., Sell D. (2024). How differences in anatomy and physiology and other aetiology affect the way we label and describe speech in individuals with cleft lip and palate. Int. J. Lang. Commun. Disord..

[6] Paradowska-Stolarz A., Mikulewicz M., Duś-Ilnicka I. (2022). Current concepts and challenges in the treatment of cleft lip and palate patients—A comprehensive review. J. Pers. Med..

[7] Lethaus B., Grau E., Kloss-Brandstätter A., Brauer L., Zimmerer R., Bartella A.K., Hahnel S., Sander A.K. (2021). Clinical follow-up in orofacial clefts—Why multidisciplinary care is the key. J. Clin. Med..

[8] Frederick R., Hogan A.C., Seabolt N., Stocks R.M.S. (2022). An ideal multidisciplinary cleft lip and cleft palate care team. Oral Dis..

[9] Mossey P.A. (2023). Global perspectives in orofacial cleft management and research. Br. Dent. J..

[10] Vieira K.M., Menezes V.C., Cardoso-dos-Santos A.C., Franco A.L.M.M., Carvalho F.M.D., Faccini L.S., Iser B.P.M. (2025). Orofacial clefts in newborns in Brazil: A time series study, 2010–2021. Epidemiol. Serv. Saúde..

[11] Silva R.S., Macari S., Dos Santos T.R., Werneck M.A., Pinto R.D.S. (2022). The panorama of cleft lip and palate live birth in Brazil: Follow-up of a 10-year period and inequalities in the health system. Cleft Palate Craniofac. J..

[12] Calderon M.G., Simoni V.C.O., Ferreira B.G.S., de Moraes A.F., Gomes M.A., Hatakeyama V.S., Santos E.F.S. (2024). Epidemiologic Characteristics, Time Trend, and Seasonality of Orofacial Clefts in São Paulo State, Brazil. 2008–2019. Cleft Palate Craniofac. J..

[13] Shibukawa B.M.C., Rissi G.P., Higarashi I.H., Oliveira R.R.D. (2020). Factors associated with the presence of cleft lip and/or cleft palate in Brazilian newborns. Rev. Bras. Saúde Mater. Infant..

[14] Ferrari-Piloni C., Barros L.A.N., Jesuíno F.A.S., Valladares-Neto J. (2021). Prevalence of cleft lip and palate and associated factors in Brazil’s Midwest: A single-center study. Braz. Oral Res..

[15] Urményi G.L., Fernandes E.C., Urményi L.G. (2024). Prevalence of cleft lip and palate in Brazil and its notification in the information system. Rev. Bras. Cir. Plást..

[16] Abreu M.H.N.G., Lee K.H., Luquetti D.V., Starr J.R. (2016). Temporal trend in the reported birth prevalence of cleft lip and/or cleft palate in Brazil, 2000 to 2013. Birth Defects Res. A Clin. Mol. Teratol..

[17] Brasil. Instituto Brasileiro de Geografia e Estatística (IBGE) Panorama. https://cidades.ibge.gov.br/brasil/panorama.

[18] Brasil. Sistema de Informações sobre Nascidos Vivos (SINASC). http://tabnet.datasus.gov.br/cgi/tabcgi.exe?sinasc/cnv/nvuf.def.

[19] Silva H.P.V.D., Arruda T.T.S., Souza K.S.C.D., Bezerra J.F., Leite G.C.P., Brito M.E.F.D., Luchessi A.D., Bortolin R.H., Ururahy M.A.G., Rezende A.A.D. (2018). Risk factors and comorbidities in Brazilian patients with orofacial clefts. Braz. Oral Res..

[20] Sousa G.F.T.D., Roncalli A.G. (2021). Factors associated with the delay in primary surgical treatment of cleft lip and palate in Brazil: A multilevel analysis. Cienc. Saude Colet..

[21] Santana B.E.F., Andrade A.C.S., Muraro A.P. (2023). Trend of incompleteness of maternal schooling and race/skin color variables held on the Brazilian Live Birth Information System, 2012–2020. Epidemiol. Serv. Saúde..

[22] Gomes J.A., Cardoso-dos-Santos A.C., Bremm J.M., Alves R.S., Bezerra A.B., Araújo V.E., Neto D.L., Cardoso L.O., Schuler-Faccini L., da Silva C.H. (2025). Congenital anomalies in Brazil, 2010 to 2022. Rev. Panam. Salud Pública..

[23] Monlleó I.L., da Silva Monteiro G., Machado J.C., de Barros A.G.R., de Andrade A.K.M., de Oliveira Júnior G.V., de Melo Brito G., Bergamini L.L., do Nascimento D.L.L., Fontes M.I.B. (2017). Fendas orais no Sistema Único de Saúde–Alagoas: Definição de modelo para referência e contrarreferência em genética. Comun. Ciênc. Saúde.

[24] Lin Y., Shu S., Tang S. (2014). A case-control study of environmental exposures for nonsyndromic cleft of the lip and/or palate in eastern Guangdong, China. Int. J. Pediatr. Otorhinolaryngol..

[25] Zhou X., Jiang Y., Fang J., Wang H., Xie D., Kuang H., Li T., Liu Q., He J. (2023). Incidence of cleft lip and palate, and epidemiology of perinatal deaths related to cleft lip and palate in Hunan Province, China, 2016–2020. Sci. Rep..

[26] da Silva A.M., Freitas V.S., Vieira A.R. (2025). Comparative study of individuals born with orofacial clefts in the United States and Brazil. J. Neonatal-Perinat. Med..

[27] Cosme H.W., Lima L.S., Barbosa L.G. (2017). Prevalence of congenital anomalies and their associated factors in newborns in the city of São Paulo from 2010 to 2014. Rev. Paul. Pediatr..

[28] Dvivedi J., Dvivedi S. (2012). A clinical and demographic profile of the cleft lip and palate in Sub-Himalayan India: A hospital-based study. Indian J. Plast. Surg..

[29] Vu G.H., Warden C., Zimmerman C.E., Kalmar C.L., Humphries L.S., McDonald-McGinn D.M., Jackson O.A., Low D.W., Taylor J.A., Swanson J.W. (2022). Poverty and risk of cleft lip and palate: An analysis of United States birth data. Plast. Reconstr. Surg..

[30] Figueiredo R.F., Figueiredo N., Feguri A., Bieski I., Mello R., Espinosa M., Damazo A.S. (2015). The role of the folic acid to the prevention of orofacial cleft: An epidemiological study. Oral Dis..

[31] Rocha C.S.M., de Souza Rocha F., Gerk A., Salomão S.L., Kim A., Telles L., Lima B.L.P., de Carvalho M.M., Alonso N. (2025). Underreporting, prevalence, and epidemiological trends of orofacial clefts in the Brazilian Amazon region. J. Craniofac. Surg..

[32] Ludorf K.L., Benjamin R.H., Canfield M.A., Swartz M.D., Agopian A.J. (2025). Prediction of preterm birth among infants with orofacial cleft defects. Cleft Palate Craniofac. J..

[33] da Silva A.M., Freitas V.S., Vieira A.R. (2025). Orofacial cleft and poor birth health outcomes: A populational cross-sectional study. Am. J. Perinatol..

[34] da Silva A.M., De Lavôr J.R., Freitas V.S., Vieira A.R. (2024). Risk of orofacial clefts in relation to maternal body mass index, diabetes and hypertension. J. Neonat. Perinat. Med..

[35] Panamonta V., Pradubwong S., Panamonta M., Chowchuen B. (2015). Global birth prevalence of orofacial clefts: A systematic review. J. Med. Assoc. Thai..

[36] Wang D., Zhang B., Zhang Q., Wu Y. (2023). Global, regional and national burden of orofacial clefts from 1990 to 2019: An analysis of the Global Burden of Disease Study 2019. Ann. Med..

[37] Slavec L., Karas Kuželički N., Locatelli I., Geršak K. (2022). Genetic markers for non-syndromic orofacial clefts in populations of European ancestry: A meta-analysis. Sci. Rep..

[38] Gomes M.B., Gabrielli A.B., Santos D.C., Pizarro M.H., Barros B.S., Negrato C.A., Dib S.A., Porto L.C., Silva D.A. (2018). Self-reported color-race and genomic ancestry in an admixed population: A contribution of a nationwide survey in patients with type 1 diabetes in Brazil. Diab. Res. Clin. Pract..

[39] Azulay R.S.D.S., Porto L.C., Silva D.A., Tavares M.D.G., Reis R.M.D.F., Nascimento G.C., Damianse S.d.S.P., Rocha V.C.d.C., Magalhães M., Rodrigues V. (2021). Genetic ancestry inferred from autosomal and Y chromosome markers and HLA genotypes in Type 1 Diabetes from an admixed Brazilian population. Sci. Rep..

[40] Gomes M.B., Rodrigues V., Santos D.C., Bôas P.R.V., Silva D.A., de Sousa Azulay R.S., Dib S.A., Pavin E.J., Fernandes V.O., Junior R.M.M. (2023). Association between HLA class II alleles/haplotypes and genomic ancestry in Brazilian patients with type 1 diabetes: A nationwide exploratory study. Genes.

